# GRP78 blockade overcomes intrinsic resistance to UBA1 inhibitor TAK-243 in glioblastoma

**DOI:** 10.1038/s41420-022-00950-5

**Published:** 2022-03-28

**Authors:** Xu Zhang, Runqiu Wu, Cong Tian, Wanzhou Wang, Lingni Zhou, Tongxuan Guo, Jiefeng Yu, Changyong Wu, Yang Shen, Xuejiao Liu, Rutong Yu

**Affiliations:** 1 Nanjing Medical University, Nanjing, 211166 Jiangsu China; 2 Insititute of Nervous System Diseases, Xuzhou Medical University, Xuzhou, Jiangsu China; 3 Department of Neurosurgery, the Affiliated Hospital of Xuzhou Medical University, Xuzhou, Jiangsu China

**Keywords:** CNS cancer, Cell death

## Abstract

Glioblastoma multiforme (GBM) is the most aggressive malignant primary brain tumor of the central nervous system. Despite continuous progression in treatment options for GBM like surgery, radiotherapy, and chemotherapy, this disease still has a high rate of recurrence. The endoplasmic reticulum (ER) stress pathway is associated with chemotherapeutic drug resistance. The UBA1 inhibitor TAK-243 can induce strong ER stress. However, the sensitivity of TAK-243 varies greatly in different tumor cells. This study evaluated the antitumor effects of the GRP78 inhibitor, HA15, combined with TAK-243 on GBM in the preclinical models. HA15 synergistically enhanced the sensitivity of GBM cells to TAK-243. When compared with TAK-243 monotherapy, HA15 combined with TAK-243 significantly inhibited GBM cell proliferation. It also induced G2/M-phase arrest in the cell cycle. In vivo studies showed that HA15 combined with TAK-243 significantly inhibited the growth of intracranial GBM and prolonged survival of the tumor-bearing mice. Mechanistically, HA15 and TAK-243 synergistically activated the PERK/ATF4 and IRE1α/XBP1 signaling axes, thereby eventually activating PARP and the Caspase families, which induced cell apoptosis. Our data provided a new strategy for improving the sensitivity of GBM to TAK-243 treatment and experimental basis for further clinical trials to evaluate this combination therapy.

## Introduction

Glioblastoma multiforme (GBM) is the most common and malignant primary brain tumor in adults. It is also one of the human malignant tumors with the highest fatality rate. Existing treatments for GBM are based on surgery combined with postoperative radiotherapy and chemotherapy, however, GBM patients typically have <15 months median survival and <6% for a 5-year survival rate [1–3]. Despite a certain understanding of GBM pathogenesis, there is still a lack of effective targeted drugs for GBM [4]. The development of new treatment strategies and the discovery of new treatments specifically targeted for GBM are of vital importance to improve the therapeutic outcomes for these patients [2, 5].

Endoplasmic reticulum (ER) proteostasis is a dynamic equilibrium process for intracellular synthesis, correct folding, and degradation of proteins in the cells. Tumor cells are affected by both internal and external factors. Tumor cells typically have significantly increased levels of misfolded proteins and unbalanced protein hemostasis, which induces ER stress. Previous reports have shown that significant ER stress is activated in the progress of tumors. ER stress has been proven to be one of the important factors of initiation, growth, and drug resistance of tumor cells [6, 7]. It is functional disorder of the ER that leads to the accumulation of unfolded or misfolded proteins, which results in decreased protein synthesis and enhanced ER degradation. This is called unfolded protein response (UPR) [8]. ER stress activates UPR through three pathways: PERK/eIF2/ATF4, IRE1α/XBP1, and ATF6/ATF6f cascade signals [9, 10]. Multiple different research groups have reported that many regulatory proteins in the UPR pathway, such as 78-kDa glucose-regulated protein (GRP78), ATF4, and IRE1α, are able to regulate tumor cell proliferation, apoptosis, and drug resistance. Expression levels of these regulatory proteins are significantly related to prognosis [6].

After the occurrence of UPR, misfolded proteins are transported to the cytoplasm and are degraded, mainly by the ubiquitin-proteasome system (UPS) [11]. The UPS is a primary cellular mechanism responsible for regulating degradation of cellular proteins and maintaining intracellular homeostasis. It contains two processes: ubiquitin modification and proteasome degradation. Ubiquitin-activating enzyme 1 (UBA1) is the main E1 enzyme [12, 13]. It activates ubiquitin and plays an important role in initiating the ubiquitination cascade. Numerous studies have reported that targeting UBA1 exhibits good tumor-suppressor effects in leukemia, liver cancer, and other tumors [14–17]. TAK-243, a first-in-class inhibitor of UBA1 [12], is currently being studied in multiple phase 1 clinical trials that focus on advanced malignancies. The UBA1 inhibitor TAK-243 can induce strong ER stress and UPR. However, the sensitivity of TAK-243 varies greatly in different tumor cells. GRP78 is a switch that controls the initiation of UPR. In other tumors, the downregulation of GRP78 has been found to increase the sensitivity of drug-resistant cells in vivo and in vitro to chemotherapeutic drugs [18–21].

In this study, we further investigated the effect of GRP78 inhibitor HA15 combined with TAK-243 on the growth of GBM cells and GBM xenografts. Targeting GRP78 enhanced the inhibitory activity of TAK-243 and promoted TAK-243-induced apoptosis of GBM cells, thereby suppressing the growth of GBM cells.

## Results

### HA15 combined with TAK-243 synergistically inhibited GBM cell viability

**Fig. 1:**
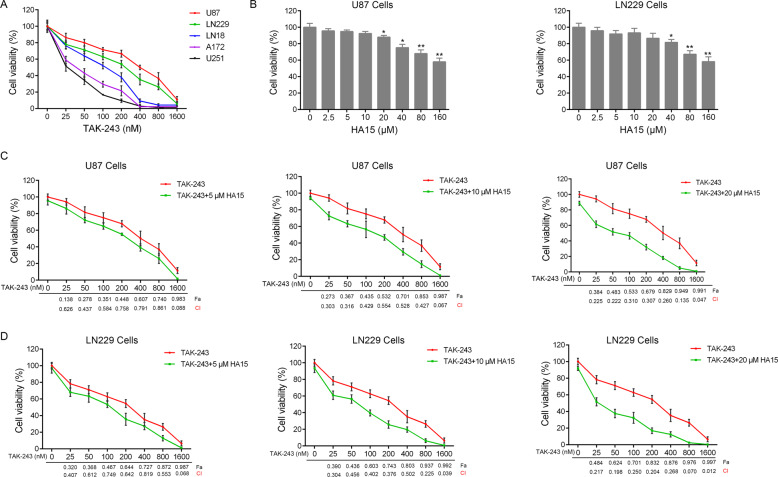
HA15 combined with TAK-243 synergistically inhibits GBM cell viability. **A** GBM cells were treated by indicated concentrations of TAK-243 for 72 h. The cell viability was examined by CCK-8 assay. **B** U87 and LN229 cells were incubated with indicated concentrations of HA15 for 72 h, respectively. Cell viability was assessed by CCK-8 assay. **C**, **D** U87 (**C**) and LN229 (**D**) cells were treated by TAK-243 combined with different concentrations of HA15 for 72 h. The cell viability was examined by CCK-8 assay. Combination index (CI) of the combination of HA15 and TAK-243 in U87 and LN229 cells was calculated by the Chou-Talalay methods.

The inhibitory effect of UBA1 inhibitor TAK-243 was evaluated in five GBM cell lines. Our results showed that TAK-243 had the worst inhibitory activity on the U87 and LN229 cells relative to other GBM cell lines (Fig. 1A). Therefore, the U87 and LN229 cell lines were used for follow-up experiments.

In order to evaluate whether the GRP78 inhibitor HA15 sensitized the inhibitory activity of TAK-243 on U87 and LN229 cells, the CCK-8 assay was used to detect the effect of HA15 + TAK-243 on the viability of U87 and LN229 cells. The results showed that when compared with TAK-243-monotherapy, HA15 + TAK-243 combination therapy significantly increased the inhibitory effect of TAK-243 on the cell viability of U87 and LN229 cells. This showed a concentration-dependent inhibition (Fig. 1C, D). However, treatment of U87 and LN229 cells with 5, 10, or 20 μM of HA15 did not show obvious inhibitory effect on cell viability (Fig. 1B). The Chou-Talalay method was further used to analyze the combined effects of these two inhibitors and to calculate the combination index (CI) of the two drugs. This is shown in Fig. 1C, D. The CI value of HA15 + TAK-243 was <1, which indicated that combination of these two compounds had a synergistic effect, and HA15 synergistically enhanced the activity of TAK-243.

### HA15 enhanced the inhibitory effect of TAK-243 on GBM cell proliferation and clone formation

**Fig. 2:**
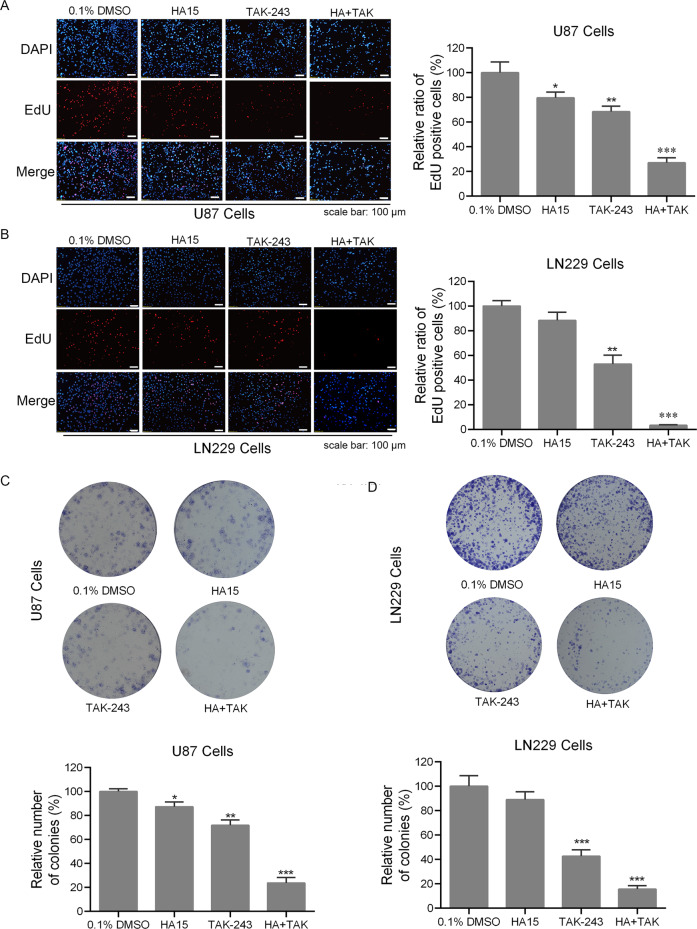
HA15 enhanced the inhibitory effect of TAK-243 on proliferation and colony formation of GBM cells. **A**, **B** Measurement of antiproliferation effects of TAK-243 (100 nM) and/or HA15 (10 μΜ) by EdU incorporation assays. Quantitative results of EdU incorporation assay were analyzed. The number of proliferative cells were normalized to that of the control group. Scale bar: 100 μm. **C**, **D** U87 and LN229 cells were treated with different concentrations of TAK-243 (100 nM) and/or HA15 (10 μΜ) for 24 h, and then changed with drug-free medium for another 12 days. The numbers of colony formation were counted. The numbers of colony formation were normalized to the control group. All the data were presented as means ± SD from three independent experiments (*P < 0.05, **P < 0.01, ***P < 0.001).

To further analyze the effect of HA15 + TAK-243 on the proliferation of GBM cells, U87 and LN229 cells were treated with 100 nM of TAK-243 and/or combined with 10 μM HA15. EdU incorporation assay was used to detect cell proliferation. This is shown in Fig. 2A, B, compared with the vehicle group, upon TAK-243 treatment, the proliferation rates of U87 and LN229 cells were reduced to 68.44% and 52.96% on average, respectively. Under co-treatment with 10 μM of HA15, the cell proliferation rates of U87 and LN229 cells were reduced to 27.69% and 3.36% on average, respectively. In order to observe the effect of HA15 + TAK-243 combination therapy on the long-term proliferation of GBM cells, a clonogenic assay was used to evaluate the ability of colony formation of the cells. Our results showed that HA15 + TAK-243 combination therapy significantly inhibited the colony formation of GBM cells, with the formation rate of 23.79% and 15.67% in the U87 and LN299 cell lines on average, respectively (Fig. 2C, D). In short, these findings indicated that HA15 enhanced the inhibitory effect of TAK-243 on the proliferation of GBM cells.

### HA15 enhanced cell cycle arrest and TAK-243-induced apoptosis of GBM cells

**Fig. 3:**
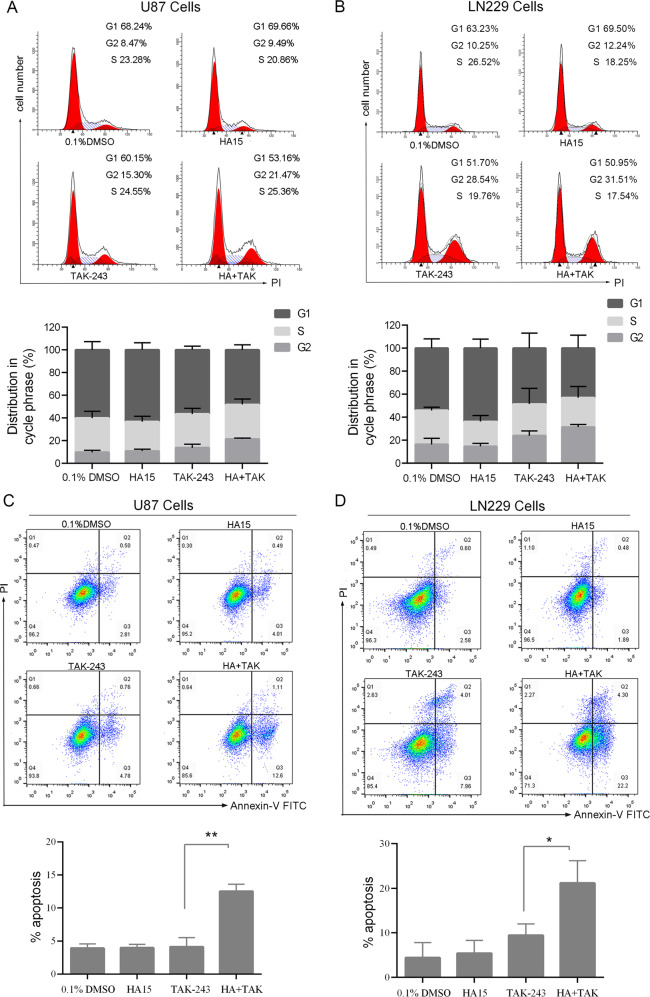
HA15 enhanced TAK-243-induced cell cycle arrest and cell apoptosis. **A**, **B** Representative data of the cell cycle analysis of TAK-243 and/or HA15-treated groups. U87 and LN229 cells were treated with TAK-243 (100 nM) and/or HA15 (10 μΜ) for 24 h. Cell cycle profile was evaluated using flow cytometry. Quantitative analysis of cell cycle phase distribution in the control group and the HA15 and/or TAK-243-treated groups. **C**, **D** U87 and LN229 cells were treated with TAK-243 (100 nM) and/or HA15 (10 μΜ) for 24 h, and were stained with Annexin V/PI. Apoptosis was evaluated by flow cytometry. The total proportion of Annexin V-positive (early apoptotic) cells and Annexin V/PI-stained double positive (late apoptotic) cells was counted as apoptotic to prepare the bar graph. The data from three independent experiments were presented as the means ± SD (*P < 0.05, **P < 0.01).

To further study the mechanism of HA15 + TAK-243 combination therapy on inhibiting GBM cell proliferation, flow cytometry was used to detect the effects of combination therapy on the cell cycle and apoptosis. Compared with TAK-243-monotehrapy, HA15 + TAK-243 combination therapy increased the proportion of GBM cells in G2/M phases. However, no significant differences in the proportion of cell cycle phase were found between the HA15-monotherapy and the vehicle groups (Fig. 3A, B). Compared with TAK-243-monotehrapy group, the apoptosis induced by TAK-243 was significantly increased after the combined usage of HA15 in treatment (Fig. 3C). The same effect was observed in LN229 cells as well (Fig. 3D). These findings indicated that HA15 + TAK-243 more obviously blocked the cell cycle process and promoted apoptosis of GBM cells.

### Inhibition of GRP78 enhanced TAK-243-induced ER stress and UPR in GBM cells

**Fig. 4:**
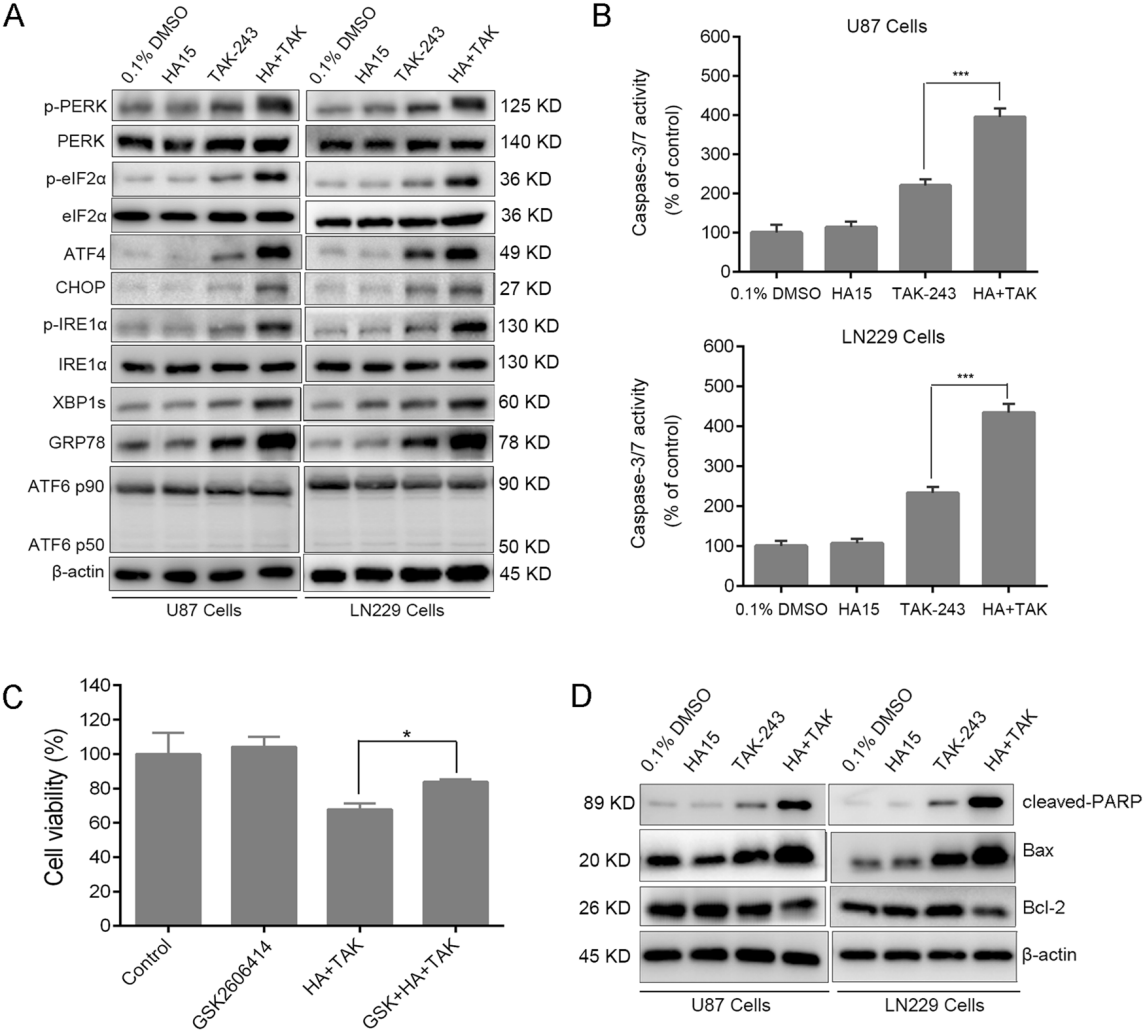
GRP78 inhibition enhances TAK-243-induced ER stress and UPR in GBM cells. **A** Representative western blot analysis showing the effects of TAK-243 combined with HA15 on the ER stress and UPR in U87 and LN229 cells. **B** U87 and LN229 cells were treated with TAK-243 (100 nM) and/or HA15 (10 μΜ) for 24 h. Caspase 3/7 activity were assessed by Caspase-Glo 3/7 activity assay. **C** U87 cells were treated with indicated inhibitors for 12 h, and then cell viability was assessed by CCK-8 assay. **D** The protein levels of cleaved PARP, Bax and Bcl-2 were assessed by immunoblotting in U87 and LN229 cells treated by TAK-243 and/or HA15. All the data were presented as means ± SD. *P < 0.05, ***P < 0.001.

We further explored the effect of HA15 + TAK-243 combination therapy on the induction of ER stress and UPR in GBM cells. Western blotting was used to detect the protein expression of molecules involved in the three different pathways related to UPR activation. The results showed that when compared with the vehicle group, HA15 had no significant effect on UPR activation at a concentration of 10 µM. TAK-243-monotherapy increased the protein expression of GRP78, p-PERK, p-EIF2a, ATF4, CHOP, p-IRE1a, and Xbp1s, while the protein expression of full-length ATF6 and cleaved ATF6 remained unchanged (Fig. 4A).

HA15 + TAK-243 combination therapy significantly enhanced the UPR effect induced by TAK-243. Compared with the TAK-243-monotherapy group, the accumulation of GRP78 of the HA15 + TAK-243 combination therapy group was increased together with the phosphorylation of PERK and eIF2a. This further induced the expression of ATF4 and CHOP, which suggested that the PERK axis was activated more significantly after the combination therapy. No effect on the expression of ATF6 was found after the combination therapy, which indicated that HA15 + TAK-243 did not affect the activation of the ATF6 axis (Fig. 4A).

Since the overactivation of UPR induced apoptosis, this study further evaluated the effect of HA15 + TAK243 combination therapy on the expression of cleaved-PARP, Bax and Bcl-2 and the activity of caspases 3/7 in GBM cells. As shown in Fig. 4D, HA15 + TAK-243 combination therapy significantly increased the expression of cleaved PARP and Bax and decreased the expression of Bcl-2 in GBM cells. The activity of cleaved caspases 3/7 was significantly increased (Fig. 4B), which was consistent with the results shown in Fig. 3C, D. To investigate the role of UPR activation in TAK-243-induced cell death, we assessed the cytotoxicity of HA15 and TAK-243 after treatment with inhibitor of UPR activation. These results showed that PERK inhibitor GSK2606414 could partly rescue HA15 combined with TAK-243 induced cell death (Fig. 4C). Taken together, inhibition of GRP78 enhanced TAK-243-induced UPR activation.

### HA15 + TAK-243 combination therapy slowed down the growth of orthotopic xenografts

**Fig. 5:**
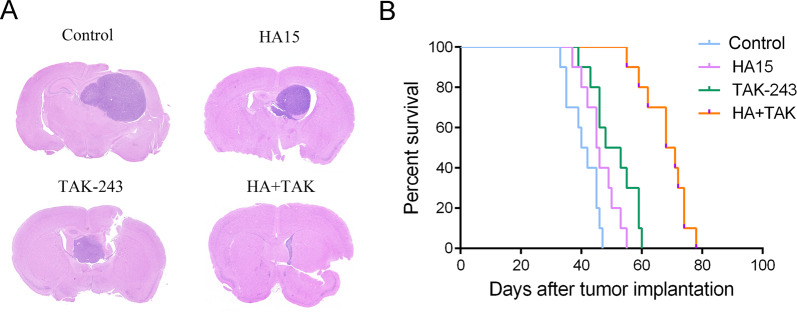
HA15 combined with TAK-243 can slow down the growth of xenograft tumor in nude mice and prolong the survival of tumor-bearing mice. **A** Mice bearing U87-drived xenograft tumor were treated with TAK-243 and/or HA15. Representative images of H&E staining of whole brain sections from control group and TAK-243 and/or HA15 treatment groups. **B** The survival analysis of mice with TAK-243 and/or HA15 treatment.

In order to further confirm the efficacy of HA15 + TAK-243 combination therapy for GBM, we constructed a U87 orthotopic xenograft GBM model in nude mice and performed different drug treatments. The hematoxylin and eosin staining showed that TAK-243-monotherapy and HA15-monotherapy inhibited the growth of U87 cells in nude mice, as compared with the vehicle group. HA15 + TAK-243 combination therapy showed a better inhibitory effect and also slowed the growth of U87 cells in the orthotopic tumors (Fig. 5A). Results of survival analysis showed that the tumor-bearing nude mice had prolonged median survival time after HA15 + TAK-243 combination therapy (Fig. 5B). Our results of in vivo experiments were consistent with those in vitro, indicating that combined targeting of GRP78 and UBA1 had a more effective antitumor effect on GBM.

## Discussion

GBM is a malignant and fatal disease among brain tumors [3]. Unfortunately, there is still a lack of effective treatment measures for GBM. The efficacy of tumor treatment is largely related to chemotherapy resistance, which has become the bottleneck of existing tumor chemotherapy regimens. In this study, we evaluated the preclinical efficacy of TAK-243 combined with HA15 in the treatment of GBM. Our results showed that HA15 synergistically increased the sensitivity of GMB cells to TAK-243. HA15 + TAK-243 combination therapy activated GBM cells to produce a stronger UPR, thereby increasing TAK-243-induced apoptosis in GBM cells.

The ER stress response is a series of compensatory protective responses initiated by cells in order to adapt to both internal and external environmental changes. Under ER stress, the sensitivity of tumor cells to chemotherapeutic drugs is significantly reduced, indicating that ER stress may be one of the important causes of multidrug resistance in tumor cells [22, 23]. The UBA1 inhibitor TAK-243 induces strong ER stress and has a good therapeutic effect on various tumors [12, 24]. However, the sensitivity of TAK-243 varies greatly in different tumors. Because TAK-243 is undergoing multiple clinical trials, it is very important to understand the mechanism of its drug resistance. A recent study has shown that inactivation of Schlafen family member 11 more effectively activates checkpoint kinase 1. This action blocks DNA synthesis and increase the sensitivity of TAK-243 [25]. It has been shown that knockout of BEN domain-containing protein 3 gene (*BEND3*) upregulates the expression of multidrug resistance protein. This results in an increase in TAK-243 efflux and reducing its binding to UBA1, thereby reducing the inhibition of UBA1 [26]. As a molecular chaperone, GRP78 has multiple functions and participates in the folding and transport of proteins. It is also a stress protein on the ER [21, 27]. Recent studies have shown that GRP78 is highly expressed in a variety of tumor cells and has a certain correlation with chemotherapy resistance [18, 19, 21, 28]. In this study, TAK-243 combined with the GRP78 inhibitor more effectively suppressed the proliferation of GBM cells. In vivo experiments further confirmed that HA15 + TAK-243 combination therapy improved the therapeutic outcomes of GBM and significantly prolonged the survival of the tumor-bearing mice. Thus, GRP78 may be a promising biomarker for TAK-243 to treat tumor sensitivity and to guide precise TAK-243 treatment for GBM.

The UPR has a dual function in tumor cells: initially compensating for damage and repairing the homeostasis of the ER. When encountering excessive or long-lasting ER stress, the cells turn from the pro-survival stage to the pro-apoptotic stage, which leads to further cell death through apoptosis [10, 29]. It has been found that UPR signaling pathways (IREI/ASK1/JNK and PERK/ATF4/CHOP) initiated by IRE1 and PERK may be involved in the induction of apoptosis [9, 10, 30, 31]. Continuous activation of PERK results in upregulation of CHOP by ATF4. Overexpression of CHOP upregulates the pro-apoptotic protein, Bim, and downregulates the major anti-apoptotic protein, Bcl-2. CHOP also upregulates the BH3-only proteins, which regulate the activation of Bax or Bak and trigger apoptosis [32, 33]. In this study, our results also showed that HA15 + TAK-243 combination therapy more significantly activated the PERK/ATF4/CHOP and IREI/XBP1s pathways than TAK-243-monotherapy. The increase in the expression level of the pro-apoptotic protein cleaved PARP and the increase in the activity of caspase 3/7 indicated that the overactivation of UPR activated the PARP and caspase families. This finally induced apoptosis and inhibited the growth of GBM cells. Thus, the combined targeting of GRP78 and UBA1 may be a more promising potential treatment strategy for GBM.

Overall, this study further demonstrated that targeting GRP78 enhanced the sensitivity of GBM cells to TAK-243. HA15 enhanced the inhibitory effect of TAK-243 on the proliferation of GBM cells by more effectively activating the UPR signaling pathway along with the PARP and caspase families to increases apoptosis. In vivo experimental results indicated that HA15 + TAK-243 combination therapy more effectively prolonged the survival of GBM-bearing nude mice and inhibited the growth of GBM cells better than HA15- or TAK-243-monotherapy. Considering these results, this study provided a new solution for improving the sensitivity of GBM to TAK-243 drugs and provided a scientific basis for further clinical trials to evaluate this combination therapy.

## Materials and methods

### Culture of cell lines

The human GBM cells lines (U87, U251, A172, LN229, and LN18) used in this study were purchased from Shanghai Cell Bank, Chinese Academy of Sciences. These cell lines were cultured and maintained in Dulbecco’s modified Eagle’s medium (DMEM) supplemented with 10% fetal bovine serum (FBS) and were grown in a humidified incubator containing 5% CO_2_ at 37 °C.

### Antibodies and reagents

PERK (#5683), eIF2α (#5324), p-eIF2α (#9721), CHOP (#2895), IRE1α (#3294), XBP1s (#27901), GRP78 (#3177), cleaved PARP (#9532), Bax (#2772), Bcl-2 (#2872) and β-actin (#9562) primary antibodies were purchased from Cell Signaling Technology (CST, MA, US). p-PERK (DF7576) antibody was purchased from Affinity Biosciences (Cincinnati, OH, USA). ATF6 (sc-166659) antibody was obtained from Santa Cruz Biotechnology (Santa Cruz, CA, US). ATF4 (ab184909) and p-IRE1α (ab243665) were purchased from Abcam (Burlingame, CA, US). UBA1 inhibitor TAK-243 was obtained from CSNpharm (CSNpharm, Chicago, IL, US). GRP78 inhibitor HA15 and PERK inhibitor GSK2606414 were purchased from MedChemExpress (Shanghai, China). TAK-243, HA15 and GSK2606414 were dissolved in DMSO to create a 10 mmol/L solution, which was diluted to different concentrations in DMEM medium before use.

### Cell viability assay

LN229 and U87 cells were inoculated into each well of a 96-well plate (4000 cells per well), with 3 replicate wells per group. After overnight incubation, different concentrations of TAK-243 and/or HA15 were added into each well for 72 h of treatment, followed by adding 10 µl cell counting kit-8 (CCK-8) solution. Samples were incubated in the dark for 30 min before the absorbance was detected at 450-nm wavelength with a microplate reader.

### Clonogenic assay

LN229 and U87 cells were inoculated into each well of 6-well plates (600 cells per well). After the cells were adhered to the wall, the cells were added with TAK-243 and/or HA15 in the culture medium for 24 h of treatment. This was followed by incubation with drug-free medium and continued culturing for 12 days. After washing with phosphate buffer saline (PBS), the cells were fixed with 4% paraformaldehyde for 30 min and stained with crystal violet working solution overnight. Cells were washed, observed, photographed, and clones were counted.

### EdU incorporation assay

The Cell-Light^TM^ EdU Cell Proliferation Detection Kit was used to detect cell proliferation according to the manufacturer’s instructions. The LN229 or U87 cell line was inoculated into each well of 96-well plate. After cell adhesion, the cells were incubated with 100 nM TAK-243 and/or 10 mM HA15 for 24 h of treatment, followed by incubating with 50 μΜ EdU for 2 h and fixing with paraformaldehyde for 30 min. After this, cells were washed with PBS and lysed with 0.5% Triton X-100 for 10 min. Cells were further incubated with 1 × Apollo^®^ reaction cocktail Kit for 30 min, and then nuclear-counterstained in the dark with DAPI solution for 15 min. After three washes in PBS, the cells were then photographed under a fluorescent inverted microscope.

### Cell cycle and apoptosis analyses

The LN229 or U87 cells were plated on 6 cm culture dishes. After cell adhesion, each dish was added with 100 nΜ TAK-243 and/or 10 μM HA15 in 0.1% DMSO for 24 h of treatment. The cells were subsequently harvested for cell cycle analysis after fixing in 70% ice-cold ethanol overnight, washing twice with PBS, and staining with propidium iodide (PI)/RNase staining solution for 15 min. For the apoptosis assay, cells were washed twice with cold PBS and stained with Annexin V-fluorescein isothiocyanate/PI apoptosis detection kit. This was followed by flow cytometry. Results were analyzed with flow-cytometry software to determine the cell cycle distribution and apoptosis.

### Caspase 3/7 activity assay

The LN229 or U87 cells were inoculated in a 96-well plate and treated with TAK-243 and/or HA15 for 24 h. Samples were then subjected to the measurement of caspase-Glo 3/7 enzymatic activities according to the manufacturer’s protocol (Promega). Briefly, 100 μl caspase-Glo 3/7 reagent was added to samples, and the solution was gently mixed well. After 30 min of incubation, 200 μl of solution from each well containing different groups of samples was transferred into a white-walled, multi-well, luminometer plate for the measurement of luminescence using the GloMax Luminometer (Promega).

### Western blotting

After treating the LN229 or U87 cells with TAK-243 and/or HA15 for 12 h, the total protein of the cells was extracted for Western blotting as described in a previous study [34]. The following specific antibodies were used for Western blotting: anti-cleaved-PARP, Bcl-2, Bax, PERK, p-PERK, eIF2, p-eIF2, ATF4, CHOP, IRE1α, p-IRE1α, XBP1s, and ATF6. Anti-β-actin antibodies were used as the loading control.

### Animal experiments

In this study, U87 cells were injected into the right striatum of nude mice in situ with a small-animal stereotaxic instrument as described in a previous study [35]. Each nude mouse was injected with 5 × 10^5^ tumor cells. Five days after tumor cell inoculation, the animals were randomly divided into four groups, with (*n* = 13 for each group). Groups were given the corresponding drug treatments: vehicle group, TAK-243-monotherapy group (10 mg/kg, intraperitoneal injection, twice a week), HA15-monotherapy group (5 μL of 100 μM HA15 in PBS per mouse, intracranial injection, once a week), and HA15 combined with TAK-243 therapy group. After 4 weeks, three tumor-bearing nude mice from each group were randomly selected and euthanized, followed by perfusion to collect the brain and hematoxylin and eosin staining to monitor tumor size. The remaining ten nude mice in each group were used for survival analysis. Mice were euthanized upon manifestation of neurological symptoms such as rotational behavior, reduced activity, dome head and so on caused by tumor progression.

### Statistical analysis

In this study, each experiment was independently repeated three times and figures shown are data from representative experiments. The GraphPad Prism 6.0 (GraphPad, San Diego, CA) software was used for statistical analysis of the experimental results. Data were presented as mean ± SD. Comparison between two samples was performed using *t* test. The Kaplan–Meier method was used for the survival analysis of the animals. With α = 0.05 as the test level, all results with *P* < 0.05 were considered to be a statistically significant difference.

## Supplementary information

Supplemental Information

## Data Availability

The datasets supporting the conclusions of this article are included within the article.
