# Supplementary material for: GRP78 blockade overcomes intrinsic resistance to UBA1 inhibitor TAK-243 in glioblastoma
Source: Cell Death Discov. 2022 Mar 28;8:133. doi: 10.1038/s41420-022-00950-5 (PMC8960808; doi:10.1038/s41420-022-00950-5)

Original Western bolts

Fig.4A U87 cells

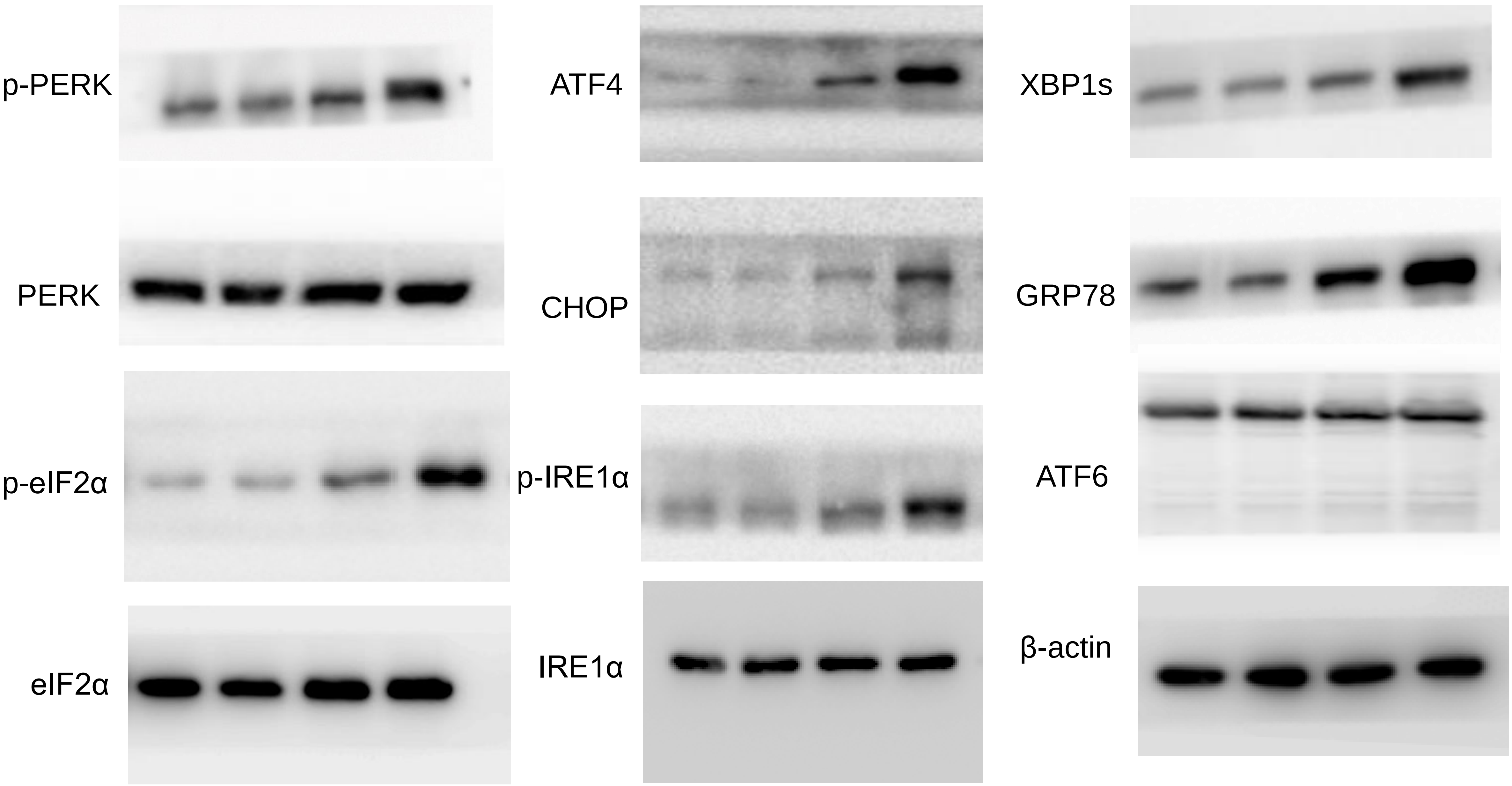

Fig.4A LN229 cells

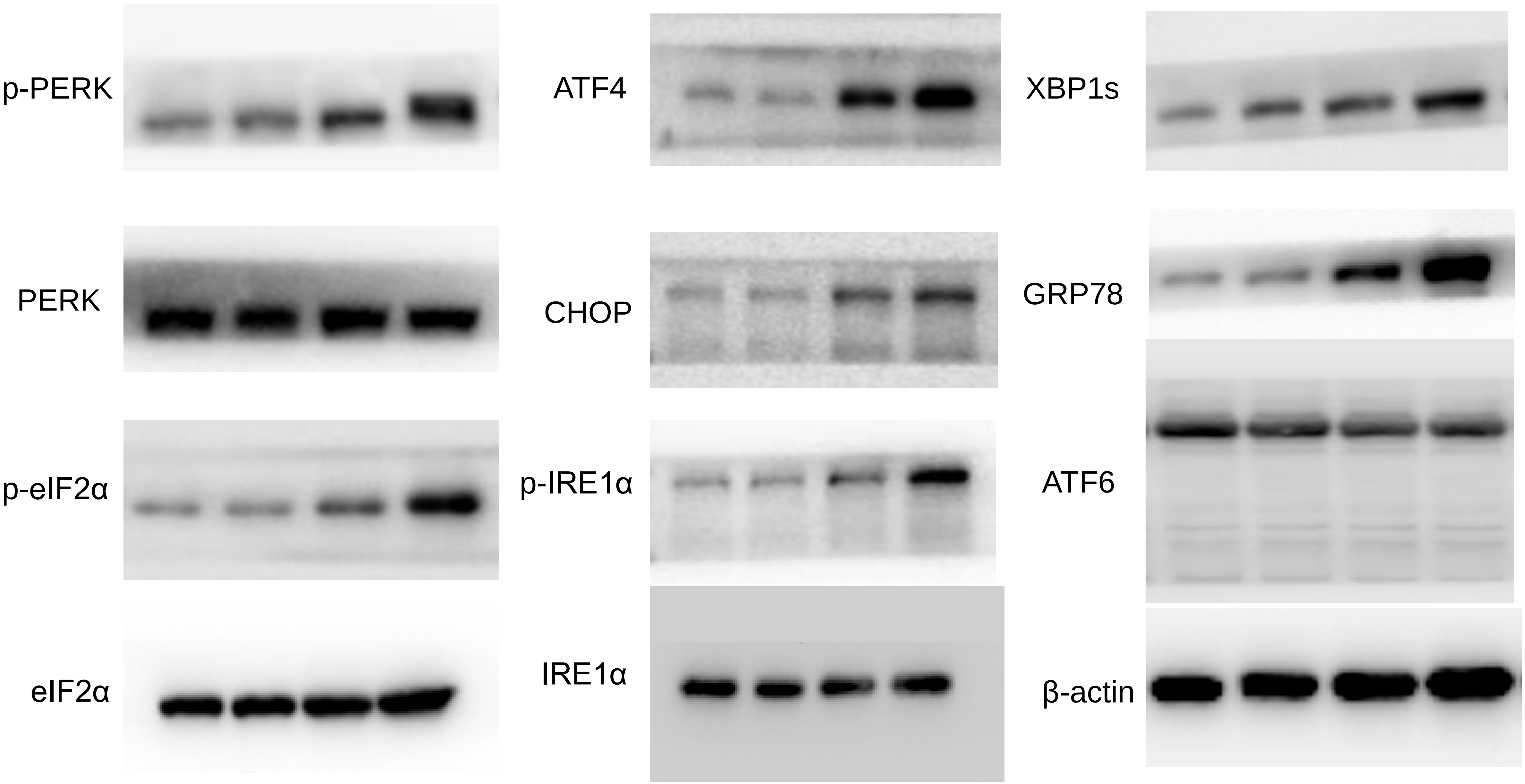

Fig.4G U87cells

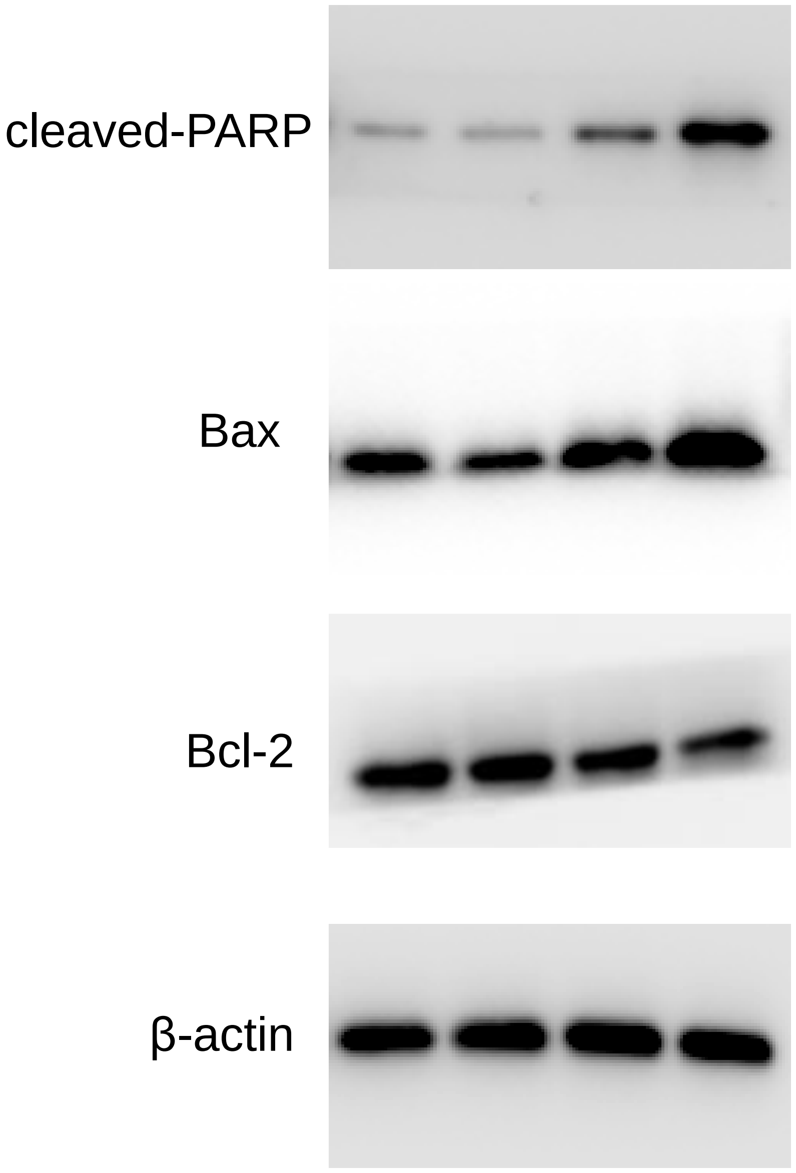

Fig.4G LN229 cells

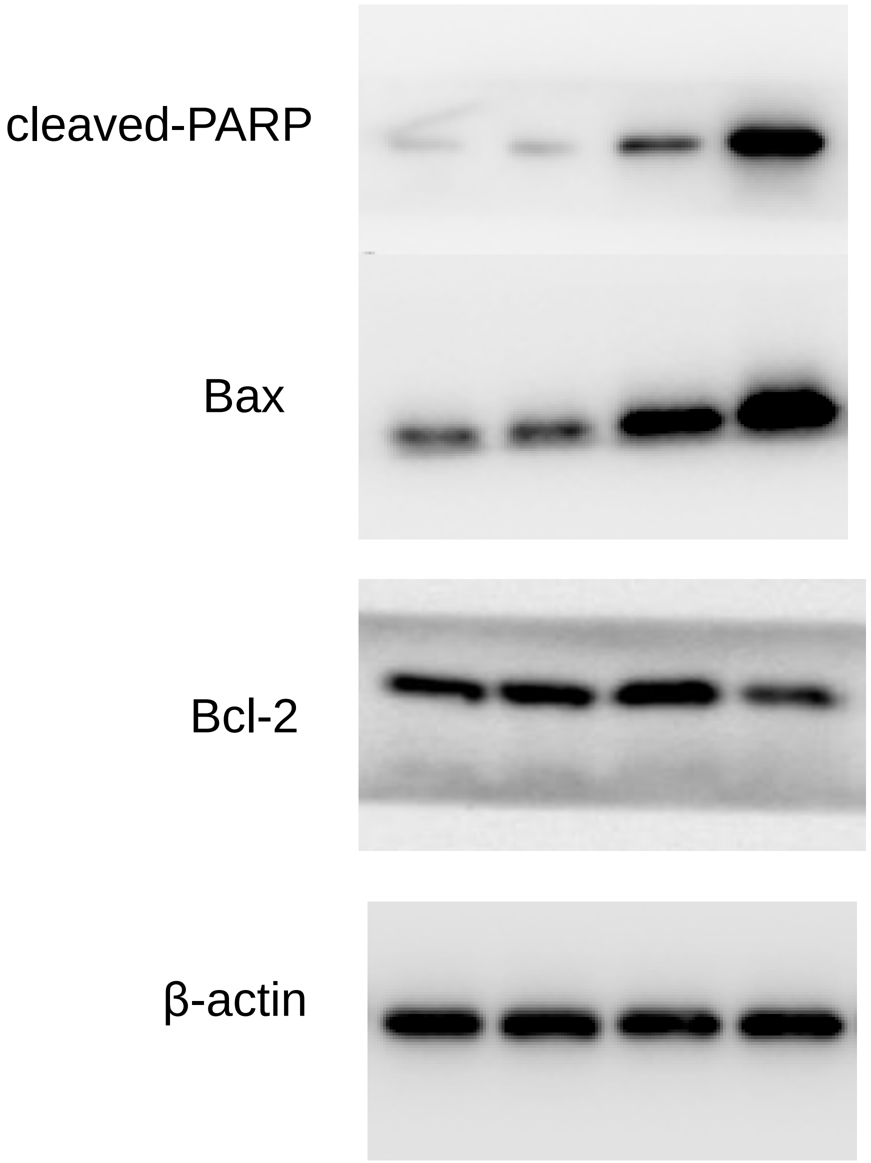

Supplement: Supplementary file 1 — Supplemental Information [file 41420_2022_950_MOESM1_ESM.pdf]
